# Language experience matters for the emergence of early numerical concepts

**DOI:** 10.1038/s41539-023-00202-w

**Published:** 2023-12-09

**Authors:** Stacee Santos, Hiram Brownell, Marie Coppola, Anna Shusterman, Sara Cordes

**Affiliations:** 1 Department of Psychology & Neuroscience, Boston College, Chestnut Hill, MA 02467 USA; 2 Departments of Psychological Sciences & Linguistics, University of Connecticut, Storrs, CT 06269 USA; 3 Psychology Department, Wesleyan University, Middletown, CT 06459 USA

**Keywords:** Human behaviour, Education

## Abstract

Research has shown a link between the acquisition of numerical concepts and language, but exactly how linguistic input matters for numerical development remains unclear. Here, we examine both symbolic (number word knowledge) and non-symbolic (numerical discrimination) numerical abilities in a population in which access to language is limited early in development—oral deaf and hard of hearing (DHH) preschoolers born to hearing parents who do not know a sign language. The oral DHH children demonstrated lower numerical discrimination skills, verbal number knowledge, conceptual understanding of the word “more”, and vocabulary relative to their hearing peers. Importantly, however, analyses revealed that group differences in the numerical tasks, but not vocabulary, disappeared when differences in the amount of time children had had auditory access to spoken language input via hearing technology were taken into account. Results offer insights regarding the role language plays in emerging number concepts.

## Introduction

Evidence suggests that language experience is the keystone for numerical development. For example, several studies have shown that the quantity and quality of number language spoken by caregivers in the home or in preschool has been found to predict children’s later performance on numerical and math assessments^1–5^, suggesting that exposure to language—particularly experience with number talk - may play a critical role in the emergence of numerical abilities early in life. Yet, other findings indicate that certain language *abilities* may relate to the acquisition of numerical concepts. For example, studies have found that vocabulary explains unique variance in the growth of numeracy scores in preschoolers^6,7^. Moreover, preschoolers’ receptive and expressive vocabularies are strongly correlated with number word acquisition (i.e., counting abilities^8^). And, in cultures lacking words for exact numbers above two or four, individuals struggle with numerical tasks involving large exact quantities^9,10^.

While this research clearly provides solid evidence for a critical role of language in early number concepts, we are left to wonder *how* this process works. Is it possible that access to language, especially number words, is the catalyst for the mind to attend to and organize numerical information, facilitating the acquisition of number concepts? Or does language play a more complex role in building number concepts, fueling a developmental cascade that fosters broader conceptual knowledge?

Here, we contribute to the breadth of literature informing our understanding of language and numerical competence. One way to unravel the complexity of the number-language relationship is to examine the emergence of numerical concepts in deaf and hard of hearing (DHH) children. DHH refers to individuals with mild to severe or profound hearing loss in one or both ears^11^. Notably, over 95% of DHH children are born to hearing parents who do not have knowledge of a signed language^12^. Because it can take several months, at minimum to be fit with hearing aids or cochlear implants, DHH children who will eventually rely on oral/spoken language and not a sign language (hereafter referred to as “oral DHH children”) typically experience an extended period of language deprivation early in development. This delayed access to language in turn results in oral DHH children falling behind in language development, particularly overall vocabulary (e.g.^13–15^).

What is less well-known is that oral DHH children also fall behind their hearing peers (HP) on mathematics abilities. Specifically, when compared to same aged HP, oral DHH school-aged children show lags in the acquisition of *symbolic* number abilities (language and symbol based, e.g., Arabic numerals) such as number knowledge^16,17^, counting (rote, one-to-one correspondence, subitizing, estimation, more/less, etc.), geometry (shape identification, sorting, mental rotation, puzzles), measurement (time, ordering length, weight, volume), and problem solving^18^, and also underperform on standardized assessments in mathematics (^19,20^, see ref. ^21^ for a review). In addition, recent research suggests that oral DHH school-aged children who are older also fall behind their same-aged HP in numerical discrimination acuity—that is, the ability to rapidly judge which of two sets of items has a greater number^22,23^. This is important because numerical discrimination abilities have been linked to success in symbolic mathematical tasks and early counting, especially the acquisition of cardinality (e.g.^24–28^). Yet the relationship between language development and non-symbolic numerical discrimination is still an open question^29^.

Why is early deafness often associated with challenges with numeracy? A handful of studies reveal that native signers—DHH children who are born to DHH parents who are fluent in sign language—perform comparably to hearing peers in math assessments, suggesting that it is unlikely that deafness itself causes difficulties in numeracy. For example, DHH children exposed to sign language from birth perform comparably to normed math assessments of hearing peers^20^ and native signing DHH adults perform comparably to hearing adults when performing arithmetic problems^30^. Though more work with native signers is needed, the data currently suggest there is more likely a language-dependent explanation for the difference in numerical abilities observed between oral DHH children and HP.

While age-related hearing loss in older adults is associated with poorer cognitive abilities (e.g.^31–34^), we do not believe these populations to be comparable. DHH children born to hearing parents experience language deprivation as they have reduced auditory access to language early in development, whereas older adults with hearing loss experience a progressive loss of auditory access later in life, after full and complete language access early in development. Thus, we argue that it is the lack of language early in development that appears to be the critical factor in the cognitive profile of DHH children. As noted, oral DHH children typically experience a period of language deprivation early in development prior to receiving hearing technology, and they can experience limitations in their access to language even after receiving such technology. In addition, oral DHH children tend to have smaller vocabularies and perhaps learn new words differently than their HP^13–15,35^. As such, it is unknown whether vocabulary, or language access more generally, might drive delays in numerical understanding.

Moreover, given that oral DHH children tend to fall behind their HP in overall vocabulary, it is likely that they may similarly fall behind in their linguistic understanding of words used to compare quantity, such as “more”. Negen and Sarnecka^36^ argue a conceptual understanding of “more” is critical for performance on numerical discrimination tasks that ask children to indicate which of two sets has “more” comparatively (see also ref. ^37^). Research has demonstrated that hearing children conceptualize the word “more” as a numerical comparative around 3 years of age^37,38^, though whether oral DHH children acquire this understanding on a similar timeline is unknown. As one specific test of the role of language ability in numerical development, we considered when oral DHH and hearing children acquire a conceptual understanding of the word “more”, and how this understanding^38^ might relate to performance on numerical discrimination tasks. We expected that competence (or lack thereof) with “more” as a linguistic comparative would be reflected in numerical discrimination performance.

In the current study, we attempt to distinguish the relative contributions of language experience (duration of auditory stimulation and thus access to linguistic input) and language abilities (specifically vocabulary) to numerical cognition in oral DHH children, and to explore whether possible delays in the understanding of the linguistic qualifier “more” in oral DHH children can account for group differences in non-symbolic numerical acuity. To do this, we first characterize early emerging symbolic (verbal “more”; number word knowledge) and non-symbolic (language-independent numerical discrimination) numerical abilities in oral DHH preschoolers and their HP (ages 3–6; *M* = 4.5 years). Number word knowledge was assessed via the Give-a-Number (Give-N) task^39^, which requires children to create a set of N objects, beginning with 1 and working up to 8 items. Performance is measured as the largest set of N objects the child can produce correctly. Understanding of “more” was assessed by presenting children cards with different numbers of items and asking them to identify which set had “more [items]”. Numerical discrimination was assessed via a computerized numerical discrimination task (see ref. ^24^) which asked children to repeatedly judge which of two arrays had the greater number of dots.

Critically, in addition to characterizing numerical abilities in oral DHH preschoolers compared to their HP, we also provide a quantitative test of potential mechanisms that may be driving their numerical delays. Namely, we investigate whether the duration of auditory stimulation and subsequent access to language a child has had, as well as the child’s general vocabulary, can account for performance differences in numerical tasks. To address differences in access to spoken language, we determined a Hearing Age for each DHH child, computed as the cumulative amount of time a child has had hearing devices, and thus maximal access to fluent language (from when the child first received auditory stimulation via hearing amplification until the date of testing) and used this measure to replace Chronological (birth) Age in our models. This allowed us to determine whether differences in the amount of time a child experienced auditory stimulation and access to language (Hearing Age) accounts for group disparities in numerical abilities between oral DHH and hearing children. In addition, we assessed vocabulary (using a parent report measure, the Developmental Vocabulary Assessment for Parents, or DVAP^40^) and entered this vocabulary measure into our models to explore how differences in acquired language account for group differences in numerical abilities.

If the emergence of numerical abilities is dependent on language access, then the amount of exposure to language (as indexed by Hearing Age) should account for the variance in performance on number measures. Further, if number learning is essentially a sub-domain of general vocabulary acquisition, then acquired vocabulary should fully account for variation in performance on number tasks. On the other hand, if number learning is more complex than simple vocabulary development, then acquired vocabulary might not account for variance in performance on the number tasks.

Together, this study aims to 1) characterize symbolic (number knowledge) and non-symbolic (number discrimination) numerical abilities and conceptual understanding of “more” in oral DHH preschoolers and their hearing peers, while 2) providing a first test of how differences in auditory language access (hearing age) and abilities (vocabulary) in these populations may account for numerical delays in oral DHH children.

## Results

### Data analyses

We used conventional multiple regression analyses^41^, with all predictors entered at the same step. Degrees of freedom differ across analyses because of incomplete data for some comparisons. In addition to conventional multiple regression analyses, we use Bayesian linear regression models for all continuous variables (conducted in JASP^42^) to provide insight into strength of the findings and speak to the reasonableness of accepting the null hypothesis (e.g., the absence of an effect of Group once Hearing age is included as an independent variable). Bayesian models have a different, useful perspective over frequentist analyses (for overviews, see refs. ^43–45^). For these analyses, we report the notation BF which represents BF_10_, the Bayes Factor for the alternate hypothesis, implying how much more likely the data are under the alternate model relative to the null model. For example, a BF = 15 indicates that the data are 15 times more likely under the alternate model rather than the null model. Bayesian statistics do not use traditional significance cut-offs (i.e., *p* < 0.05), rather the value of the Bayes Factor itself is considered evidence, or strength, for the alternate model. The general interpretation of the strength of the evidence based on the Bayes Factor is: BF < .10: Strong Evidence for the null hypothesis; BF = .10 - .33: Substantial Evidence for the null hypothesis; BF - .33 - .99: Anecdotal Evidence for the null hypothesis; BF = 1: No evidence for either hypothesis; BF = 1–3: Anecdotal Evidence for the alternative hypothesis; BF = 3–10: Substantial Evidence for the alternative hypothesis; BF = 10–30: Strong Evidence for the alternative hypothesis; BF = 30–100: Very Strong Evidence for the alternative hypothesis; BF > 100: Decisive Evidence for the alternative hypothesis^46^. For models with multiple effects (i.e., age, group, or vocabulary), we report BF_incl_ for each effect as support for the inclusion of the variable in the model. Note that all Bayesian findings align with results of our frequentist statistics—they are included to provide greater support for our analyses.

All Bayesian analyses used JASP default priors reflecting the Cauchy priors with widths of 0.707 (which can be interpreted as 50% certainty that the effect is between −0.707 and 0.707 for the two-sided hypothesis^47^. Previous literature suggests that default priors be used in numerical cognition unless there is strong prior knowledge for the effect sizes^48^.

#### Group differences across tasks: language experience matters

##### Summary

To characterize group differences (see Table 1), we subjected each dependent variable (Verbal “more”, Numerical Discrimination, Number Knowledge, and Vocabulary) to separate conventional multiple regression analyses^41^ with the child’s Chronological Age and Group (DHH or HP) as predictors. Both Number Knowledge and Verbal “More” data were somewhat dichotomous due to ceiling effects. Thus, alternative analyses involving logistic regressions were performed, coding performance as 100% vs. <100%. Results were the same using both approaches to the analysis. We then completed the same analyses using Bayesian linear regression models in order to obtain Bayes Factors. Results revealed Group differences across all measures, such that DHH children performed lower compared to hearing peers across all four measures. Bayesian analyses revealed at least Substantial Evidence (*BF* > 3) in favor of Chronological Age and Group as strong predictors of performance (Table 2). Individual regression analyses reported below:

**Table 1:** Descriptive Statistics Means (SD).

	DHH	HP
Chronological Age (months)	54.62 m (12.26)	55.07 m (13.09)
Hearing Age (months)	41.10 m (12.39)	–
Verbal “More” (total score)	9.60 (3.25)	11.20 (2.20)
Numerical Discrimination (% correct)	69.36 (20.54)	82.63 (15.95)
Counting Knowledge (knower level)	4.07 (2.3)	5.18 (1.43)
Vocabulary (total score)	63.43 (24.48)	113.64 (35.40)

**Table 2:** Results of Linear Regression and Bayesian Analyses with Group and Chronological Age.

	*β*	*t*	*p*	*BF*_*incl*_	*R*^*2*^	*F*	*df*	*p*	*BF*_*model*_
Verbal “More”					0.260	10.04	2, 57	<0.001	BF_10_ = 149.13
Chron Age	0.428	4.97	**<0.001**	88.51					
Group	−0.272	−2.39	**0.020**	5.85					
Numerical discrimination					0.349	14.99	2, 56	<0.001	BF_10_ = 3255.71
Chron Age	0.497	4.61	**<0.001**	1219.30					
Group	−0.317	−2.94	0.**005**	18.54					
Number knowledge					0.338	14.31	2, 56	<0.001	BF_10_ = 68791.37
Chron Age	0.512	4.712	**<0.001**	36855.29					
Group	−0.263	−2.42	**0.019**	9.33					
Vocabulary					.400	15.68	2, 47	<0.001	BF_10_ = 3269.98
Chron Age	0.286	2.52	**0.015**	6.98					
Group	−0.534	−4.70	**<0.001**	1313.27					

Bold values indicates statistical significant (*p* > .05).

#### Verbal “More”

Results revealed significant group differences for the verbal “more” measure with oral DHH children performing lower than their HP, F(2,57) = 10.04, *p* < 0.001, (*BF* = 149.13), with Chronological Age (*β* = 0.428, *p* < 0.001; *BF*_*incl*_ = 88.51) and Group (*β* = −0.272, *p* = 0.020; *BF*_*incl*_ = 5.85) both contributing to the overall model.

#### Numerical discrimination

Oral DHH children performed significantly worse than their HP, F(2,56) = 14.99, *p* < 0.001, (*BF* = 3255.71), on the numerical discrimination task, with Chronological Age (*β* = 0.497, *p* < 0.001; *BF*_*incl*_ = 1219.30) and Group (*β* = −0.317, *p* = 0.005; *BF*_*incl*_ = 18.54) both contributing to the overall model.

#### Number knowledge

Again, results revealed oral DHH children had significantly lower number knowledge scores than their HP, F(2,57) = 14.31, *p* < 0.001 (*BF* = 68,791.37), with Chronological Age (*β* = 0.512, *p* < 0.001; *BF*_*incl*_ = 36,855.29) and Group (*β* = −0.263, *p* = 0.019; *BF*_*incl*_ = 9.33) both contributing to the overall model.

#### Vocabulary

Consistent with the numerical measures, results revealed significant group differences in vocabulary scores with oral DHH children performing lower than their HP, F(2,47) = 15.68, *p* < 0.001, (*BF* = 3269.98) with Chronological Age (*β* = 0.286, *p* = 0.015; *BF*_*incl*_ = 6.98) and Group (*β* = −0.534, *p* < 0.001; *BF*_*incl*_ = 1313.27) both contributing to the overall model.

Then, to explore whether differences in early language access may account for these group differences, a second set of conventional multiple regression analyses were completed, replacing Chronological Age with Hearing Age, such that Hearing Age and Group were the only predictors of performance on the four dependent measures. The bivariate correlation between Hearing age and Group was approximately *r* = 0.6 across the many regression models. Diagnostics for multicollinearity revealed variance inflation factors ranging from 1.00 to 1.76 indicating no multicollinearity concerns^49,50^. Because Hearing Age and Chronological Age are collinear (*r* = 0.84), we could not include both in the regression model with our sample size. However, the above analyses show that Group differences are not accounted for by Chronological Age. The critical question for the next set of analyses was whether Hearing Age can explain the observed differences between DHH children and their HP. If so, then performance would be explained by the duration of a child’s language experience.

Overall, results revealed that Hearing Age significantly predicted performance for each dependent measure, and the main effect of Group disappeared for three measures: Verbal “More”, Numerical Discrimination, and Number Knowledge. In line with these findings, Bayesian analyses revealed Decisive Evidence (*BF*_*incl*_ > 100) in favor of Hearing Age as a strong predictor of performance, with No Evidence (*BF*_*incl*_ < 1) that Group explained performance (Table 3). Individual regression analyses are reported below:

**Table 3:** Results of linear regression and bayesian analyses with group and Hearing Age.

	*β*	*t*	*p*	*BF*_*incl*_	*R*^*2*^	*F*	*df*	*p*	*BF*_*model*_
Verbal “More”					0.283	11.06	2, 56	<0.001	BF_10_ = 280.35
Hearing Age	0.513	4.12	**<0.001**	504.52					
Group	−0.063	−0.339	0.736	0.50					
Numerical discrimination					0.356	15.18	2, 55	< .001	BF_10_ = 3497.39
Hearing Age	0.583	4.91	**<0.001**	6382.32					
Group	−0.031	−0.265	0.792	0.43					
Number knowledge					0.408	18.95	2, 55	<0.001	BF_10_ = 2.450e + 6
Hearing Age	0.650	5.66	**<0.001**	5.442e + 6					
Group	0.024	0.213	0.832	0.36					
Vocabulary					0.433	17.56	2, 46	<0.001	BF_10_ = 8358.37
Hearing Age	0.414	3.24	**0.002**	38.19					
Group	−0.345	−2.70	**0.010**	10.15					

Bold values indicates statistical significant (*p* > .05).

#### Verbal “More”

When Hearing Age replaced Chronological Age in the model, results revealed no Group differences for the verbal “more” measure with oral DHH children performing comparably to their HP, F(2,56) = 11.06, *p* < 0.001, (*BF* = 280.35), with Hearing Age (*β* = 0.513, *p* < 0.001; *BF*_*incl*_ = 504.52) but not Group (*β* = − 0.063, *p* = 0.736; *BF*_*incl*_ = 0.50) contributing to the overall model.

#### Numerical discrimination

When Hearing Age was entered as a predictor, Group no longer significantly predicted performance on the numerical discrimination task. The overall model was significant F(2,55) = 15.18, *p* < 0.001, (*BF* = 3497.39), with Hearing Age (*β* = 0.583, *p* < 0.001; *BF*_*incl*_ = 6382.32) but not Group (*β* = −0.031, *p* = 0.792; *BF*_*incl*_ = 0.434) contributing to the overall model.

#### Number knowledge

Again, Group differences disappeared when Hearing Age was entered into the model predicting Number knowledge F(2,55) = 18.95, *p* < 0.001, (*BF* = 2.450e + 6), with Hearing Age (*β* = 0.650, *p* < 0.001; *BF*_*incl*_ = 5.44e + 6) but not Group (*β* = 0.024, *p* = 0.832; *BF*_*incl*_ = 0.364) contributing to the overall model.

#### Vocabulary

Unlike performance on the numerical measures, results of analyses with Hearing Age and Group predicting vocabulary performance continued to reveal significant group differences in vocabulary scores with oral DHH children performing lower than their HP, F(2,46) = 17.56, *p* < 0.001, (*BF* = 8358.37) with Hearing Age (*β* = 0.414, *p* = 0.002; *BF*_*incl*_ = 38.19) and Group (*β* = −0.345, *p* = 0.010; *BF*_*incl*_ = 10.15) both contributing to the overall model.

**Fig. 1:**
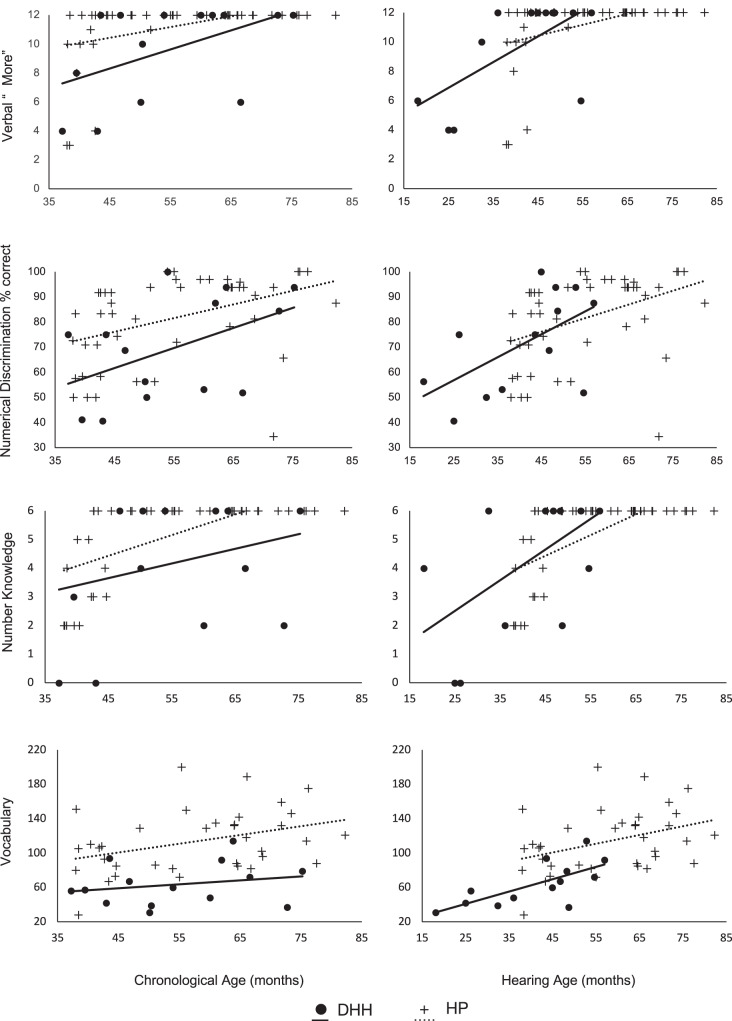
Performance on Verbal “More”, Numerical Discrimination, Number Knowledge, and Vocabulary as a function of Group and Chronological age (on the left) and as a function of Group and Hearing age (on the right). Crosses represent Hearing children’s performances, and filled dots represent DHH children’s performances. Best fitting regression lines for Chronological age and Group (dotted line: Hearing children and solid lines: DHH children).

Thus, including our measure of auditory language access diminished any group differences found in our three measures of numerical ability but not in vocabulary (Fig. 1).

#### Auditory language experience vs. language abilities (Vocabulary)

As expected, Hearing Age correlated strongly with Vocabulary measures, *r*(46) = 0.59, *p* < 0.001; the longer a child has had access to fluent language, the longer a child has been able to acquire new vocabulary. As such, we wanted to know whether the duration of language access *or* the amount of acquired vocabulary was the more important determinant of children’s numeracy skills. To explore the contribution of vocabulary ability to performance on numerical tasks, we performed conventional multiple regression analyses followed by Bayesian linear regression analyses with Hearing Age, Group, and Vocabulary as predictors of performance on the three numerical dependent measures (Table 4).

**Table 4:** Results of Linear Regression and Bayesian Analyses with Hearing Age, Group, and Vocabulary.

	*β*	*t*	*p*	*BF*_*incl*_	*R*^*2*^	*F*	*df*	*p*	*BF*_*model*_
Verbal “More”					0.343	7.84	3, 45	<0.001	BF_10_ = 117.96
Hearing Age	0.455	2.95	**0.005**	30.66					
Group	0.044	0.290	0.773	0.49					
Vocabulary	0.219	1.36	0.179	0.90					
Numerical discrimination					0.388	9.29	3, 44	<0.001	BF_10_ = 379.89
Hearing Age	0.548	3.66	**<0.001**	310.32					
Group	−0.092	−0.628	0.533	0.44					
Vocabulary	0.039	0.249	0.805	0.40					
Number knowledge					0.450	12.26	3, 45	<0.001	BF_10_ = 428,357.12
Hearing Age	0.653	4.63	**<0.001**	105,866.80					
Group	0.108	0.784	0.437	0.36					
Vocabulary	0.108	0.733	0.467	0.34					

Bold values indicates statistical significant (*p* > .05).

As seen in Table 4, across all three numerical dependent measures (Verbal “More”, Numerical Discrimination, Number Knowledge), models that included Vocabulary (along with Hearing Age and Group) as a predictor of performance did not result in a significantly higher overall prediction of the model. Vocabulary was not found to be a significant predictor of performance on any of the numerical tasks. Bayesian analyses revealed Strong to Decisive Evidence in favor of Hearing Age and No Evidence for Group or Vocabulary as predictors of performance across any task.

#### Verbal “More”

Results revealed no influence of vocabulary in the verbal “more” measure, F(3,45) = 7.84, *p* < 0.001, (*BF* = 117.96.35). Hearing Age (*β* = 0.455, *p* = 0.005; *BF*_*incl*_ = 30.66) but not Group (*β* = 0.044, *p* = 0.773; *BF*_*incl*_ = 0.49) nor Vocabulary (*β* = 0.219, *p* = 0.733; *BF*_*incl*_ = 0.90) contributing to the overall model.

#### Numerical discrimination

Results revealed no influence of vocabulary in the Numerical Discrimination task, F(3,44) = 9.29, *p* < 0.001, (*BF* = 379.89), with Hearing Age (*β* = 0.548, *p* < 0.001; *BF*_*incl*_ = 310.32) but not Group (*β* = −0.092, *p* = 0.533; *BF*_*incl*_ = 0.44) or Vocabulary (*β* = 0.039, *p* = 0.805; *BF*_*incl*_ = 0.40) contributing to the overall model.

#### Number knowledge

Results revealed no influence of Vocabulary on Number Knowledge, F(3,45) = 12.26, *p* < 0.001, (*BF* = 428,357.12), with Hearing Age (*β* = 0.653, *p* < 0.001; *BF*_*incl*_ = 105,866.80) but not Group (*β* = 0.108, *p* = 0.437; *BF*_*incl*_ = 0.36) nor Vocabulary (*β* = 0.108, *p* = 0.467; *BF*_*incl*_ = 0.34) contributing to the overall model.

Again, Hearing Age was a significant predictor, while Group and Vocabulary were *not* found to predict performance. Together, these findings support our contention that language *access* (as measured by Hearing Age), rather than language ability (as measured by vocabulary), is a catalyst for refining the development of numerical abilities.

### Is understanding the word “more” important for numerical discrimination tasks?

Lastly, we explored whether performance on the Verbal “More” task impacted children’s performance on Numerical Discrimination. We performed conventional multiple regression analyses with Hearing Age, Group, and Verbal “More” scores as predictors of performance on the Numerical Discrimination task, followed by comparable Bayesian linear regression analyses (Table 5). The overall prediction here was strong, F(3,54) = 13.09, *p* < 0.001, (*BF* = 12,468.55), with Hearing Age (*β* = 0.426, *p* = 0.002; *BF*_*incl*_ = 68.12) and Verbal “More” (*β* = 0.305, *p* < 0.001; *BF*_*incl*_ = 4.61), but not Group (*β* = −0.013, *p* = 0.913; *BF*_*incl*_ = 0.603) contributing to the overall model.

**Table 5:** Results of Linear Regression and Bayesian Analyses for Numerical Discrimination with Hearing Age, Group, and Verbal “More”.

	*β*	*t*	*p*	*BF*_*incl*_	*R*^*2*^	*F*	*df*	*p*	*BF*_*model*_
Numerical discrimination					0.421	13.09	3, 54	<0.001	BF_10_ = 12,468.55
Hearing Age	0.426	3.27	**0.002**	68.12					
Group	−0.013	−0.110	0.913	0.60					
Verbal “More”	0.305	2.45	**0.018**	4.61					

Bold values indicates statistical significant (*p* > .05).

This pattern signals the importance of knowledge of quantifier language in numerical discrimination tasks. While our Verbal “More” task offered limited variability in responses (most hearing children older than 3.5 years were at ceiling), it does suggest that understanding of linguistic quantifiers may play a critical role in performance on numerical discrimination tasks. Results are the same when Verbal “More” is treated as a binary outcome. Note that, because we included a number of nonverbal practice trials reinforcing the correct selection of the greater number of items at the beginning of the numerical discrimination task, we believe that children who did not have a proficient understanding of “more” were still able to complete the numerical discrimination task. This belief is confirmed by the data—though 9 children performed below chance on the verbal more task, seven of those nine children performed above chance on the numerical discrimination task. This suggests that while there is an important connection between a conceptual understanding of the word “more” and performance on the numerical discrimination task, verbal “more” understanding was not a requirement to perform at a level greater than would be expected by chance in our numerical discrimination task.

## Discussion

It is difficult to isolate the role language itself plays in developing early numerical concepts. Nevertheless, researchers have tried to explain how language promotes numerical understanding, suggesting pivotal roles of both language *experience* and the acquisition of language *ability* in the development of numerical concepts (e.g.^1–8^). In this study we explored how both language experience (as measured by hearing age) and ability (as measured by vocabulary) contribute to the disparities in mathematics abilities historically observed between oral DHH and hearing children.

We focused on number knowledge and numerical discrimination abilities in our preschool sample because of their important connection to mathematics abilities later in life (e.g.^25,27,28,51–53^). Extending prior work on numerical delays in oral DHH children (e.g.^16–19^), we found that oral DHH children underperform compared to their HP in both symbolic (number knowledge) and non-symbolic (numerical discrimination) numerical tasks and in overall vocabulary.

Notably, this is one of just a few studies to explore early number knowledge acquisition in DHH preschool children using the standard Give-N procedure, allowing us to provide direct evidence that oral DHH preschoolers lag in their acquisition of counting and number word knowledge compared to their same aged HP (see also ref. ^17^). The Give-N procedure is considered to be a gold-standard for assessment of number knowledge in hearing preschoolers, and performance has been marked as an important predictor of future math abilities^54^. Prior work assessing counting abilities in DHH children has focused on rote counting, or pointing tasks which have been found to assess procedural knowledge—not conceptual knowledge—of early counting (e.g.^16,17^). Thus, our findings extend prior work by providing strong evidence for a lag in *conceptual* understanding of number knowledge in oral DHH preschoolers.

This was also one of the first studies to capture numerical discrimination in oral DHH preschoolers, the youngest age at which numerical discrimination in DHH children has been explored. Our results corroborate previous work that shows lower numerical discrimination abilities in older DHH children and adults^22,23^, while providing evidence that these disparities in non-symbolic numerical discrimination abilities are evident in oral DHH children as young as 3-years-old. Notably, numerical discrimination abilities have been linked to math abilities (e.g.^24^); as such, these results align with documented struggles oral DHH children experience with symbolic math at this age, prior to formal math education (e.g.^17,18^). Future work should investigate whether group differences in numerical discrimination are present even earlier in development—perhaps prior to the onset of productive language in infancy—and whether differences in language access play a role in these abilities and/or in the relationship between non-symbolic and symbolic number development in oral DHH children.

In addition, this was the first study to explore oral DHH children’s verbal understanding of “more” as a comparative quantifier. Similar to performance on the number knowledge and numerical discrimination tasks, hearing children outperformed oral DHH children in identifying “more” as a comparative quantifier. Given the importance of comparative quantifiers for the acquisition of numerical concepts (e.g., ref. ^37^), it is noteworthy that oral DHH children fall behind their HP in their understanding of this concept. That these differences disappear when Hearing Age is considered suggests that language experience sets the pace for the development of linguistic quantifiers.

It is important to note that while we do not find that the conceptual understanding of “more” as a linguistic quantifier to be necessary to complete numerical discrimination tasks, our findings indicate that it may promote refined discrimination abilities and should be a consideration when exploring numerical discrimination acuity in young children. Future research should explore performance on numerical discrimination tasks without the nonverbal priming trials to see if verbal “more” is necessary to complete the task or critical for refining discrimination abilities.

In addition to characterizing group differences in performance, we also explored the role of language in driving these performance disparities. By including DHH children born to hearing parents who consequently experienced language deprivation early in development, we were able to define a unique variable, Hearing Age, to reflect the amount of time a child has had auditory access to spoken language. This allowed us to distinguish the impact of language experience from the effect of language ability (as measured by DVAP vocabulary scores), thus exploring unique pathways language may contribute to numerical development. However, we note that we used Hearing Age as a measure of the length of time the child had received auditory stimulation, a proxy for the time they had language access, yet simultaneously acknowledge that hearing technology does not provide full and complete access to language at the level of their hearing peers. As such, our Hearing Age measure only provides an estimate of the child’s language access since birth but does not take into account the reduced access oral DHH children using auditory technology may experience on a day-to-day basis.

The most notable finding that emerged from this study is that group differences in performance disappeared when differences in the amount of time children experienced auditory access to language (Hearing Age) was entered into the model. That a DHH child that has had auditory access to language for three years performs similarly to a three-year-old hearing child on tasks assessing symbolic number knowledge, non-symbolic numerical discrimination, and verbal “more” understanding, speaks to the importance of language access for cognitive development. Remarkably, although vocabulary scores and Hearing Age were related, vocabulary knowledge was not an important contributor to group differences in the numerical tasks when Hearing Age was also included in the model. This refines previous research showing that language ability, as indicated by acquired vocabulary, sets the pace for number learning in DHH children^17^. The current findings raise the possibility that the range of experiences supported by language access may be more important than simple vocabulary when considering the effects of language on the emergence of both symbolic number knowledge and non-symbolic numerical discrimination abilities.

Importantly, our findings suggest that while vocabulary may play a role in numerical acquisition in both typically hearing^8^ and DHH children^17^, the broader experiences afforded by access to language—including exposure to number talk, access to syntax and grammatical markers for quantity^55–57^, and even more nuanced conversational cues indicating quantity that may be available to the child—may not be captured by a simple vocabulary measure. Instead, our Hearing Age variable, which measures the amount of time each child had access to these broader experiences, better captures how the accumulation of these experiences foster the acquisition of symbolic and non-symbolic numerical abilities.

Further, the inclusion of Hearing Age did not fully account for group differences in *vocabulary* between oral DHH children and their HP. This could potentially reflect lack of proficient auditory stimulation from hearing technology or a reflection of different language environments of oral DHH children—both consequently leading to DHH children not experiencing the same quality or quantity of language access. Research has shown that overall vocabulary size predicts a child’s word learning strategies^58^, and a child’s lexicon is more strongly correlated with word learning than age^59^. That oral DHH children have lower vocabularies to begin with^13–15^, there is likely a compounding effect of vocabulary knowledge that is reflected here. While vocabulary outcomes have improved in line with breakthroughs in hearing technology and more effective language rehabilitation methods over the last 50 years, oral DHH children as a group do not appear to be catching up in vocabulary development compared to their hearing peers (e.g.^13,60–63^).

Our analyses identify language access, particularly very early in development, as an important catalyst for attending to and organizing numerical information, a critical element in the development of numerical concepts. It is important to emphasize that the differences in auditory access to language between the two groups occurred in the first year or two of life-long before children were speaking or likely being introduced to rich numerical content. This early linguistic experience may set children up to learn numerical content later. How? One possibility is that language deprivation may slow the acquisition of basic language skills. Given that oral DHH children tend to have smaller vocabularies related to their HP (e.g.,^13–15,17,58–63^) and that parents tend to adjust their speech to their child’s vocabulary knowledge^64^, they may simply be exposed to numerical language later than their HP. Future work should record the linguistic environments of oral DHH children to explore this possibility. Regardless, our finding is striking as it highlights evidence that early language experience, even before the onset of expressive language, is critical for setting the stage for later numerical development.

This work suggests that language experience (duration of auditory stimulation and thus access to linguistic input) is at the root of numerical development. Understanding how language experience engages mechanisms involved in emerging number concepts is up for debate. One possibility is that greater language experience equates to more number language input which, in turn, promotes a child’s attention to numerical information in the environment. That is, when DHH children begin to have auditory access to fluent English, they are also likely to experience more number talk from their caregivers. In turn, this number talk can promote more attention to number in the world around them, sometimes called Spontaneous Focus on Number (SFON; 65). SFON has been identified as an important predictor of math ability^65^ that is sensitive to environmental variables^66^ and reduced SFON in DHH children may drive group differences in numerical tasks starting in early childhood. Future research should investigate this possibility.

Our main finding—that auditory stimulation and subsequent access to language matters more for numerical acquisition than vocabulary ability—hinges upon the fact that we used a valid measure of child vocabulary. Although the DVAP has not been used previously with oral DHH children, we have every reason to believe that parent reports using the DVAP should provide an accurate snapshot of child vocabulary in this population. In hearing samples, the DVAP has been found to be a sound alternative to traditional experimenter-administered vocabulary measures as it displays strong relationship to both the MacArthur-Bates CDI^67^, a well-known parent-report of expressive language development, and the Peabody Picture Vocabulary Test-4 (PPVT-^4,68^). Further, parent-report measures have been found to be useful tools in assessing vocabulary development in both English-speaking (oral) and signing DHH children^69^ and have even been found to be comparable to experimenter-administered assessments^70^. Together, these studies suggest that our vocabulary measure was appropriate for this sample, and thus point to language access, and not vocabulary, as an important catalyst for the emergence of numerical concepts.

In addition, we did not ask parents of the children in either group whether their child had experienced frequent ear infections, or whether they were experiencing an ear infection during the time of testing. While unlikely to meaningfully impact language access at the time of testing, reoccurring (or current) ear infections could impact language access, potentially increasing the difficulty of understanding the task. It is important for future work to consider capturing this during data collection.

While our results have emphasized the importance of language access for both symbolic and non-symbolic numerical acquisition, it is important to acknowledge the possibility that Hearing Age may be confounded with other important variables, such as socioeconomic status (SES; parent education level and/or household income) and educational access. While prior research has shown that SES is correlated with hearing preschoolers’ numerical abilities (e.g.^71,72^) as well as with DHH children’s spoken language abilities^73^, other work has not found SES to be correlated with Hearing Age or the amount of therapeutic support DHH children receive^74^. Although we did not collect demographic information about family SES in our sample, these studies suggest that it is unlikely that SES, and not Hearing Age, explain performance in our DHH participants.

Moreover, the DHH children in this study were monolingual and had minimal, if any, exposure to American Sign Language (ASL). Although this work does not speak to how DHH preschoolers learning ASL acquire numerical concepts, we would expect a similar pattern such that any group differences would be explained by the cumulative time the child was exposed to a signed language such as ASL. Accordingly, we predict that DHH children who are exposed to ASL from birth (and thus do not experience language deprivation early in development) are unlikely to demonstrate any notable delays in numerical development (see ref. ^20^). Future work should be sure to include children who use ASL and attempt to isolate the role that language experience may play in the development of numerical concepts, particularly with DHH children who are bilingual, that is, learning both a signed and a spoken language.

Finally, it is important to recognize our sample was small. Because congenital hearing loss is rare—fewer than 2 out of every 1000 infants screened in the United States were identified with a permanent hearing loss in 2019^75^—even with our intense, concentrated recruitment strategies and extensive travel, our efforts yielded a small sample of children. Further, children with unilateral and bilateral hearing loss, moderate to severe hearing loss, and who use hearing aids versus cochlear implants have different experiences regarding access to language. Unfortunately, this small sample does not allow us to explore how using different hearing devices and/or different levels of hearing may account for performance. However, level of hearing loss has not been found to be associated with performance on numerical tasks (e.g.^22^). This is an interesting question that should be explored further in future research. Nonetheless, we have provided strong evidence for the importance of language access—not ability—in the acquisition of numerical concepts in preschool oral DHH children. The secondary Bayesian analyses provide robust evidence to support these findings.

There are many illustrations demonstrating the critical role language plays in numerical cognition. Some are striking yet understandable, and others more nuanced and complicated. Here we introduce new evidence attempting to elucidate this relationship by isolating the duration of language access and exploring its impact on the emergence of early numerical abilities. This work highlights the relation between the cumulative time that children have access to language and the development of numerical concepts, specifically number knowledge and numerical discrimination, emphasizing the benefits of early, fluent language access for oral DHH children. Taken together, results from this study provide novel insight into theories about the role of language in the acquisition of foundational math abilities.

## Methods

### Participants

Fourteen (5 females; *M* = 4.5 years; SD = 1.02) oral DHH children and 46 (29 females; *M* = 4.6 years; SD = 1.08) hearing children between 3 and 6 years of age participated in the study. Our small sample of oral DHH children reflects the low prevalence of DHH children in the general population. Because of this we included three times the number of HP to increase our statistical power. All children used English as their primary form of communication.

Hearing children were recruited via study announcements from mainstream preschools and museums in the New England area. Our hearing sample included 74% White, 1% Asian, and 19% multiracial participants (6% not reported). We did not inquire about the presence of chronic otitis media with effusion (COME), a condition that presents as fluid in the middle ear, often post ear infection, or recurring presence of fluid in the middle ear without infection which can impact hearing^76^.

Oral DHH participants were primarily recruited from two deaf and hard of hearing preschools that engage in oral deaf education in the northeastern United States. One child attended a mainstream preschool (i.e., where their classmates were all hearing), and the remainder attended preschools that focused on oral and auditory language skills (no sign language use or instruction). All children were born to non-signing hearing parents. Hearing loss ranged from moderate to profound. All hearing loss data was obtained through parent report. All but one of the DHH participants had a bilateral hearing loss (additional exploratory analyses with hearing age excluding this child did not alter the pattern of results) and most were diagnosed with a permanent hearing loss at birth. Two of the fourteen DHH children were diagnosed after 12 months of age (22 and 30 months, respectively) resulting in late amplification (see Table 6 for DHH statistics). Two additional DHH participants were excluded from the analysis for lack of participation in the tasks, and because the parent indicated a progressive hearing loss identified after 4 years of age. This child consequently did not experience the early language deprivation that is commonly experienced by DHH children born to non-signing parents.

**Table 6:** Parent-report characterizations of hearing and family history for DHH sample.

*N*	14
Sidedness	
Bilateral	13
Amplification	
Hearing Aids	9
Cochlear Implants	4
None	1
Degree of hearing loss	
Mild	0
Moderate	4
Mod-Sev	4
Severe	2
Profound	4
Family history	
Parents DHH	0

### Tasks and procedures

This study was approved by the Institutional Review Board at Boston College. Parents provided written informed consent prior to participation. Participants were tested in a quiet area of their preschool, home, or the research lab. Each child completed three tasks that were administered in the following order: (1) Verbal “More” task, (2) Numerical Discrimination task, and (3) Give-N task. While children performed the tasks, all parents completed the Developmental Vocabulary Assessment for Parents (DVAP^40^) and the parents of DHH children were given an additional questionnaire regarding their child’s hearing, language exposure, and use of auditory amplification.

### Child measures

#### Verbal “More” task

This task assessed participants’ understanding of the word “more” as indicating a greater quantity. Participants were shown 12 boards (14” × 11”) displaying two sets of cards (5” × 8”) containing varying quantities of randomly arranged stickers. For each trial, children were asked, “Which card has more [objects]?” Cards contained between one and 12 stickers randomly placed on the card. The order of presentation was 2 vs. 5 (Pretzels), 3 vs. 7 (Jellyfish), 12 vs. 4 (Lions), 8 vs. 4 (Footballs), 2 vs. 6 (Fish), 3 vs. 1 (Mermaids), 9 vs. 4 (Rhinos), 10 vs. 5 (Starfish), 2 vs. 8 (Soccer balls), 12 vs. 5 (Turtles), 3 vs. 11 (Sharks), 2 vs. 3 (Taco trucks). Participants were allowed to count, but no feedback was given to the children.

#### Numerical discrimination

We used a computer program designed to measure non-symbolic numerical discrimination acuity^24–26^. The program was run on a 13” MacBook Air. During each trial, participants were shown two boxes, side-by-side on a gray background; the left box had yellow dots inside a yellow perimeter with an image of the Sesame Street character Big Bird just outside the box to the bottom left. The right box had blue dots inside a blue perimeter with an image of the character Grover just outside the box to the bottom right. On a random half of the trials, the cumulative area of the dots in each box was equated and on the other half of trials, the average size of the dots in each array was controlled.

On every trial, dots were briefly displayed (display time 2100 ms) and children were asked, “Who has more dots?” Children were instructed to point or say which box they thought had a greater number of dots. Set sizes ranged between 5 and 21 dots with ratios between 1.2 and 2.8. The experimenter sat to the side of the screen and pressed the corresponding button to reflect the child’s response. No feedback was given during the task. Given the age of the children, and prior work showing percent correct to be an appropriate measure of numerical discrimination in this task^26^, we used this as our dependent measure of numerical discrimination. This task differed from the verbal “more” task in three important ways: the numerical discrimination task presented more challenging numerical ratios, larger set sizes (outside of the small number range), and a shorter duration of stimuli presentation (too short to invoke verbal counting).

Critically, prior to completing the numerical discrimination task, participants had a warm-up task to promote their understanding of task instructions. The warm-up task was a simpler version of the numerical discrimination task (involving only 3:1 numerical ratios) which provided visual feedback for both correct (a short cartoon clip) and incorrect (a large red X) responses. Stimuli were screen captures taken from the same numerical discrimination task described above. The task continued until the child provided eight correct responses in a row or completed 40 trials (whichever came first). Initial analyses revealed that although older children required fewer trials to reach criterion (Chronological Age: beta = −0.322, *t* = −2.56, *p* = 0.013, BF_incl_ = 3.5), there were no Group differences in the number of trials to reach criterion (Group: beta = 0.168, *t* = 1.34, *p* = 0.186, BF_incl_ = 1.53).

#### Number knowledge

Children then participated in the standard Give-N task (based on refs. ^26,39^). Children were presented ten 3” yellow rubber ducks and a 9” blue circular plastic plate. The experimenter explained the task, “These are my ducks, and this (pointing to the plate) is my pond. I am going to put *one* duck into the pond like this” (experimenter places one duck into the “pond” then places it back with the set). “Can you put one duck into the pond?” (if the child puts one duck into the pond), “Is that *one* duck?” (when the child acknowledged one duck, the experimenter removed it from the “pond” and placed it with the other ducks). “Can you put *two* ducks into the pond?” (if the child places two ducks into the pond) “Is that *two* ducks?” (if the child affirms) “Can you count to make sure?”. After the child counts the ducks, the experimenter then removes the ducks from the pond and asks for a different number of ducks. If the child correctly placed N ducks into the pond and correctly counted the ducks to confirm the quantity, the experimenter continued the task with N + 1 ducks and followed the same procedure for 3, 4, 5, 6 ducks (in that order) with the addition of 7 and 8, or until the child failed to correctly place the number asked. If the child placed an incorrect number of ducks, the experimenter asked for N−1 ducks. This procedure continued until the child got N correct twice, and N + 1 incorrect twice. The final number of ducks correctly entered into the “pond” and confirmed (counted) was considered the child’s *number knower-level*. Knower-level scores were coded between 0 and 6. Though children were asked to place as many as 8 ducks into the pond, to align with conventional scoring of performance on the Give-N task (e.g.^26^), the maximum knower-level assigned to any child was 6, thus, all proficient counters were coded with a knower-level of 6 for analyses.

### Parent questionnaires

#### Vocabulary

Parents completed the Developmental Vocabulary Assessment for Parents (DVAP*;* 40) as a measure of the child’s vocabulary abilities. The DVAP is a quick, parent-administered questionnaire comprised of the first 204 words from Form A of the Peabody Picture Vocabulary Test-4 (PPVT-4; 67). The DVAP is designed to be used with children between the ages 2–7 years and found to correlate strongly with a child’s actual and future PPVT-4 scores^40^.

#### Hearing questionnaire

Parents of DHH children completed an additional questionnaire to obtain information about their child’s hearing history. From the information provided on the Hearing Questionnaire, a Hearing Age was computed for each child. Hearing Age was calculated by subtracting the age (in months) the child began accessing speech through hearing technology from the child’s chronological age at test (months). For example, if a child was 60 months at time of test and began wearing hearing aids at 20 months, her Hearing Age would be 60–20 = 40 months. One of the parents of a DHH child did not complete the hearing questionnaire, resulting in a Hearing Age for only 13 of the 14 DHH participants. For the Hearing Peer group, Hearing Age was by definition the same as chronological age.

## Data Availability

The datasets generated during and/or analyzed during the current study are deposited in the Open Science Framework repository and are available at https://osf.io/e9vqn/?view_only=f962497e00e34447a35d2992d6d97c7b.

## References

[1] Gibson, D. J., Gunderson, E. A. & Levine, S. C. Causal Effects of Parent Number Talk on Preschoolers’ Number Knowledge. *Child Dev.*10.1111/cdev.13423 (2020) 10.1111/cdev.13423 PMC10683715 33164211

[2] Gunderson EA, Levine SC (2011). Some types of parent number talk count more than others: relations between parents’ input and children’s cardinal-number knowledge. Dev. Sci..

[3] He, S. et al. Assessing Efficacy and Benefit of a Behavioral Math Talk Intervention for Caregivers of Young Children. *Child Youth Care Forum*10.1007/s10566-022-09671-3 (2022).

[4] Levine SC, Suriyakham LW, Rowe ML, Huttenlocher J, Gunderson EA (2011). Dev. Psychol..

[5] Klibanoff RS, Levine SC, Huttenlocher J, Vasilyeva M, Hedges LV (2006). Preschool children’s mathematical knowledge: The effect of teacher ‘math talk. Dev. Psychol..

[6] Purpura DJ, Hume LE, Sims DM, Lonigan CJ (2011). Early literacy and early numeracy: The value of including early literacy skills in the prediction of numeracy development. J. Exp. Child Psychol..

[7] Yang X, Dulay KM, McBride C, Cheung SK (2021). How do phonological awareness, rapid automatized naming, and vocabulary contribute to early numeracy and print knowledge of Filipino children?. J. Exp. Child Psychol..

[8] Negen J, Sarnecka BW (2012). Number-concept acquisition and general vocabulary development: general vocabulary development. Child Dev..

[9] Pica P, Lemer C, Izard V, Dehaene S (2004). Exact and approximate arithmetic in an amazonian indigene group. Science.

[10] Gordon P (2004). Numerical cognition without words: evidence from Amazonia. Science.

[11] Deafness and Hearing Loss. Retrieved from https://www.who.int/news-room/fact-sheets/detail/deafness-and-hearing-loss (2021).

[12] Mitchell RE, Karchmer MA (2004). Chasing the mythical ten percent: parental hearing status of deaf and hard of hearing students in the United States. Sign Lang. Stud..

[13] Carrigan, E. & Coppola, M. Delayed language exposure has a negative impact on receptive vocabulary skills in deaf and hard of hearing children despite early use of hearing technology. In (eds Brown, M. M. & Kohut, A.) *BUCLD 44: Proceedings of the 44th annual Boston University Conference on Language Development* (Cascadilla Press, Somerville, 2020).

[14] Lederberg AR, Schick B, Spencer PE (2013). Language and literacy development of deaf and hard-of-hearing children: successes and challenges. Dev. Psychol..

[15] Lund E (2016). Vocabulary knowledge of children with cochlear implants: a meta-analysis. J. Deaf Stud. Deaf Educ..

[16] Leybaert J, Van Cutsem M-N (2002). Counting in sign language. J. Exp. Child Psychol..

[17] Shusterman A, Peretz-Lange R, Berkowitz T, Carrigan E (2022). The development of early numeracy in deaf and hard of hearing children acquiring spoken language. Child Dev..

[18] Pagliaro CM, Kritzer KL (2013). The Math Gap: a description of the mathematics performance of preschool-aged deaf/hard-of-hearing children. J. Deaf Stud. Deaf Educ..

[19] Kritzer KL (2009). Barely started and already left behind: a descriptive analysis of the mathematics ability demonstrated by young deaf children. J. Deaf Stud. Deaf Educ..

[20] Hrastinski I, Wilbur RB (2016). Academic Achievement of Deaf and Hard-of-Hearing Students in an ASL/English Bilingual Program. J. Deaf Stud. Deaf Educ..

[21] Santos, S. & Cordes, S. Math abilities in deaf and hard of hearing children: the role of language in developing number concepts. *Psychol. Rev.*10.1037/rev0000303 (2021). 10.1037/rev0000303 34138618

[22] Bull R, Marschark M, Nordmann E, Sapere P, Skene WA (2018). The approximate number system and domain-general abilities as predictors of math ability in children with normal hearing and hearing loss. Br. J. Dev. Psychol..

[23] Ma H, Bu X, Sanford EM, Zeng T, Halberda J (2021). Approximate number sense in students with severe hearing loss: a modality-neutral cognitive ability. Front. Hum. Neurosci..

[24] Halberda J, Mazzocco MMM, Feigenson L (2008). Individual differences in non-verbal number acuity correlate with maths achievement. Nature.

[25] Libertus ME, Odic D, Feigenson L, Halberda J (2016). The precision of mapping between number words and the approximate number system predicts children’s formal math abilities. J. Exp. Child Psychol..

[26] Shusterman A, Slusser E, Halberda J, Odic D (2016). Acquisition of the cardinal principle coincides with improvement in approximate number system acuity in preschoolers. PLoS ONE.

[27] Libertus ME, Feigenson L, Halberda J (2011). Preschool acuity of the approximate number system correlates with school math ability: Approximate number system and math abilities. Dev. Sci..

[28] Starr A, Libertus ME, Brannon EM (2013). Number sense in infancy predicts mathematical abilities in childhood. Proc. Natl Acad. Sci. USA.

[29] Libertus ME, Feigenson L, Halberda J (2013). Is approximate number precision a stable predictor of math ability?. Learn Individ Differ..

[30] Berteletti, I., Kimbley, S., Sullivan, S., Quandt, L. & Miyakoshi, M. Language modality does not change how our brain distinguishes different mathematical operations. 10.31234/osf.io/3p9jr (2021)

[31] Lin FR (2011). Hearing loss and incident dementia. Arch. Neurol..

[32] Lin FR (2014). Association of hearing impairment with brain volume changes in older adults. NeuroImage.

[33] Livingston G (2020). Dementia prevention, intervention, and care: 2020 report of the Lancet Commission. Lancet.

[34] Uchida Y (2019). Age-related hearing loss and cognitive decline - The potential mechanisms linking the two. Auris, Nasus, Larynx.

[35] Lederberg, A. R. & Spencer, P. E. Vocabulary development of young deaf and hard of hearing children. In (eds Clark, M. D., Marschark, M. & Karchmer, M.) *Context, Cognition, and Deafness,* 73–92 (Gallaudet University Press, Washington DC, 2001).

[36] Negen J, Sarnecka BW (2015). Is there really a link between exact-number knowledge and approximate number system acuity in young children?. Br. J. Dev. Psychol..

[37] Odic D, Pietroski P, Hunter T, Lidz J, Halberda J (2013). Young children’s understanding of “more” and discrimination of number and surface area. J. Exp. Psychol.: Learn., Mem., Cognition.

[38] Gathercole VC (1985). More and more and more about more. J. Exp. Child Psychol..

[39] Wynn K (1990). Children’s understanding of counting. Cognition.

[40] Libertus ME, Odic D, Feigenson L, Halberda J (2015). A developmental vocabulary assessment for parents (DVAP): validating parental report of vocabulary size in 2- to 7-year-old children. J. Cognition Dev..

[41] Cohen, J., Cohen, P., West, S. G. & Aiken, L. S. (2003). Applied multiple regression/correlation analysis for the behavioral sciences, 3^rd^ edn. (Erlbaum, Mahwah, NJ, 2003).

[42] JASP team. JASP version 0.16.1. (2022).

[43] Dienes Z, Mclatchie N (2018). Four reasons to prefer Bayesian analyses over significance testing. Psychonomic Bull. Rev..

[44] Kruschke JK, Liddell TM (2018). The Bayesian New Statistics: hypothesis testing, estimation, meta-analysis, and power analysis from a Bayesian perspective. Psychonomic Bull. Rev..

[45] Wagenmakers E-J (2018). Bayesian inference for psychology. Part I: Theoretical advantages and practical ramifications. Psychon. Bull. Rev..

[46] Wagenmakers E-J, Wetzels R, Borsboom D, van der Maas HLJ (2011). Why psychologists must change the way they analyze their data: The case of psi: Comment on Bem (2011). J. Personal. Soc. Psychol..

[47] Bartlett, J. (n.d.). *An Introduction to JASP: A Free and User-Friendly … | Study notes Statistics | Docsity*. Retrieved December 14, 2022, from https://www.docsity.com/en/an-introduction-to-jasp-a-free-and-user-friendly/8999270/.

[48] Faulkenberry, T. J., Ly, A. & Wagenmakers, E.-J. Bayesian inference in numerical cognition: a tutorial using JASP. 10.31234/osf.io/vg9pw (2020)

[49] Johnston R, Jones K, Manley D (2018). Confounding and collinearity in regression analysis: a cautionary tale and an alternative procedure, illustrated by studies of British voting behaviour. Qual. Quant..

[50] Menard, S. *Applied Logistic Regression Analysis* 2nd edn. (Sage Publications, London, 2001).

[51] Chu, F. W., vanMarle, K. & Geary, D. C. Predicting children’s reading and mathematics achievement from early quantitative knowledge and domain-general cognitive abilities. *Front. Psychol*. 10.3389/fpsyg.2016.00775 (2016). 10.3389/fpsyg.2016.00775 PMC4879431 27252675

[52] Rittle-Johnson B, Zippert EL, Boice KL (2018). Data on preschool children’s math, patterning, and spatial knowledge. Data Brief..

[53] van Marle K, Chu FW, Li Y, Geary DC (2014). Acuity of the approximate number system and preschoolers’ quantitative development. Dev. Sci..

[54] Geary DC (2011). Consequences, characteristics, and causes of mathematical learning disabilities and persistent low achievement in mathematics. J. Dev. Behav. Pediatrics.

[55] Bloom P, Wynn K (1997). Linguistic cues in the acquisition of number words. J. Child Lang..

[56] Syrett K, Musolino J, Gelman R (2012). How can syntax support number word acquisition?. Lang. Learn. Dev..

[57] Almoammer A (2013). Grammatical morphology as a source of early number word meanings. Proc. Natl Acad. Sci. USA.

[58] Lederberg AR, Prezbindowski AK, Spencer PE (2000). Word-learning skills of deaf preschoolers: the development of novel mapping and rapid word-learning strategies. Child Dev..

[59] Lederberg AR, Spencer PE (2009). Word-learning abilities in deaf and hard-of-hearing preschoolers: effect of lexicon size and language modality. J. Deaf Stud. Deaf Educ..

[60] El-Hakim H (2001). Vocabulary acquisition rate after pediatric cochlear implantation and the impact of age at implantation. Int. J. Pediatr. Otorhinolaryngol..

[61] Convertino C, Borgna G, Marschark M, Durkin A (2014). Word and world knowledge among deaf learners with and without cochlear implants. J. Deaf Stud. Deaf Educ..

[62] Davidson LS, Geers AE, Nicholas JG (2014). The effects of audibility and novel word learning ability on vocabulary level in children with cochlear implants. Cochlear Implants Int.

[63] Lund E, Schuele CM (2014). Effects of a word-learning training on children with cochlear implants. J. Deaf Stud. Deaf Educ..

[64] Leung A, Tunkel A, Yurovsky D (2021). Parents fine-tune their speech to children’s vocabulary knowledge. Psychol. Sci..

[65] Hannula MM, Lepola J, Lehtinen E (2010). Spontaneous focusing on numerosity as a domain-specific predictor of arithmetical skills. J. Exp. Child Psychol..

[66] Braham EJ, Libertus ME, McCrink K (2018). Children’s spontaneous focus on number before and after guided parent–child interactions in a children’s museum. Dev. Psychol..

[67] The MacArthur-Bates Communicative Development Inventories User’s Guide and Technical Manual, Second. https://products.brookespublishing.com/The-MacArthur-Bates-Communicative-Development-Inventories-Users-Guide-and-Technical-Manual-Second-Edition-P78.aspx.

[68] Peabody Picture Vocabulary Test--Fourth Edition - PsycNET. https://psycnet.apa.org/doiLanding?doi=10.1037%2Ft15144-000.

[69] Prezbindowski AK, Lederberg AR (2003). Vocabulary assessment of deaf and hard-of-hearing children from infancy through the preschool years. J. Deaf Stud. Deaf Educ..

[70] Bornstein MH, Selmi AM, Haynes OM, Painter KM, Marx ES (1999). Representational abilities and the hearing status of child/mother dyads. Child Dev..

[71] Arnold DH, Fisher PH, Doctoroff GL, Dobbs J (2002). Accelerating math development in Head Start classrooms. J. Educ. Psychol..

[72] Jordan NC, Kaplan D, Nabors Oláh L, Locuniak MN (2006). Number sense growth in kindergarten: a longitudinal investigation of children at risk for mathematics difficulties. Child Dev..

[73] Haukedal CL (2022). Social communication and quality of life in children using hearing aids. Int. J. Pediatr. Otorhinolaryngol..

[74] Smith B (2019). Effects of socioeconomic status on children with hearing loss. Int J. Pediatr. Otorhinolaryngol..

[75] CDC. Data and Statistics About Hearing Loss in Children | CDC. *Centers for Disease Control and Prevention* https://www.cdc.gov/ncbddd/hearingloss/data.html (2021).

[76] Ear Infections in Children. https://www.nidcd.nih.gov/health/ear-infections-children (2022).

